# Dispositional mindfulness: Dissociable affective and cognitive processes

**DOI:** 10.3758/s13423-024-02462-y

**Published:** 2024-02-01

**Authors:** Nancy Tsai, Isaac N. Treves, Clemens C. C. Bauer, Ethan Scherer, Camila Caballero, Martin R. West, John D. E. Gabrieli

**Affiliations:** 1 McGovern Institute for Brain Research and Department of Brain and Cognitive Sciences, Massachusetts Institute of Technology, Cambridge, MA 02139 USA; 2 Department of Psychology, Northeastern University, 805 Columbus Avenue, Boston, MA 02139 USA; 3 Harvard Graduate School of Education, Cambridge, MA 02138 USA; 4 Department of Psychology, Yale University, 2 Hillhouse Avenue, New Haven, CT 06511 USA; 5 MIT Integrated Learning Initiative, Cambridge, MA 02139 USA

**Keywords:** Mindfulness, Cognition, Stress, Affect, Adolescents

## Abstract

Mindfulness has been linked to a range of positive social-emotional and cognitive outcomes, but the underlying mechanisms are unclear. As one of the few traits or dispositions that are associated with both affective and cognitive benefits, we asked whether mindfulness is associated with affective and cognitive outcomes through a shared, unitary process or through two dissociable processes. We examined this in adolescents using behavioral measures and also reanalyzed previously reported neuroimaging findings relating mindfulness training to either affect (negative emotion, stress) or cognition (sustained attention). Using multivariate regression analyses, our findings suggest that the relationships between dispositional mindfulness and affective and cognitive processes are behaviorally dissociable and converge with neuroimaging data indicating that mindfulness modulates affect and cognition through separate neural pathways. These findings support the benefits of trait mindfulness on both affective and cognitive processes, and reveal that those benefits are at least partly dissociable in the mind and brain.

## Introduction

Mindfulness is defined as an awareness of the present moment combined with an attitude of nonjudgment and curiosity (Bishop et al., 2004). Trait or dispositional mindfulness, as measured via self-report questionnaires (MAAS, Black et al., 2012; FFMQ, Baer et al., 2004), refers to a stable, dispositional quality of mindfulness (Brown et al., 2007), and has been correlated in adults with better psychological well-being (Cash & Whittingham, 2010), lower stress and anxiety (Arch & Craske, 2010), better cognitive function (Riggs et al., 2015), and better educational outcomes in adolescents (Caballero et al., 2019). Similarly, mindfulness training has been associated with decreased stress and negative affect (Schonert-Reichl & Lawlor, 2010) and improved cognitive performance (Cásedas et al., 2020; Chiesa et al., 2011). These findings show that greater mindfulness benefits *both* affective and cognitive functions, although the mechanisms underlying these relations is largely unknown. Here we assessed whether the associations between dispositional mindfulness and affective or cognitive functions reflect a unitary process or, instead, two dissociable processes.

Dispositional mindfulness may be associated with salutary affective outcomes by modulating affective experience. Here we define affective processes as those involved in emotions, moods, preferences, attitudes, value, and stress (Gross & Barrett, 2013). Specifically, greater mindfulness has been associated with lesser reactivity to negative affective stimuli (Guendelman et al., 2017), higher levels of positive affect, and lower levels of negative affect (Brown et al., 2012; Keng et al., 2011; Kong et al., 2014; Short & Mazmanian, 2013; Treves et al., 2022). Studies with non-clinical populations have reported that higher dispositional mindfulness is associated with fewer symptoms of depression (Barnhofer et al., 2011; Bränström et al., 2011), post-traumatic stress disorder (Smith et al., 2011), and borderline personality disorder (Fossati et al., 2011), and better overall psychological well-being (Bajaj et al., 2016). There is also neuroimaging evidence that dispositional mindfulness mediates the link between resting-state connectivity and positive affect (Kong et al., 2016).

Dispositional mindfulness may also modulate affective experiences of stress. The stress-buffering hypothesis posits that dispositional mindfulness attenuates the link between daily stress and negative affect (Dixon & Overall, 2016), and has been supported by observational research linking self-reported mindfulness in daily life to positive psychological health and lower levels of distress (Bao et al., 2015; Brown et al., 2007; Grossman et al., 2004; Keng et al., 2011; Kong et al., 2014; Ryan & Brown, 2003; Schutte & Malouff, 2011). In experimental studies where stress was manipulated, baseline dispositional mindfulness predicted lower stress reactivity (Arch & Craske, 2010; Bullis et al., 2014). One of the few studies with adolescents found that greater dispositional mindfulness in the face of stress exposure predicted fewer psychological symptoms (Cortazar & Calvete, 2019). Together, these findings highlight the links between mindfulness and multiple affective processes.

Despite the sizable literature on the affective benefits of dispositional mindfulness, less is known about the effects of mindfulness on cognition. Executive Function (EF), a collection of cognitive processes that underly goal-directed behavior, is argued to be essential during the practice of mindfulness to control and sustain attention (Chatzisarantis & Hagger, 2007; Thomson et al., 2015). Multiple studies have linked greater dispositional mindfulness to better EF performance in adults (Lyvers et al., 2014; Rosenberg et al., 2013) and adolescents (Oberle et al., 2012; Riggs et al., 2015; Shin et al., 2016). In numerous experimental studies, improved mindfulness enhanced sustained attention (Bauer et al., 2020; Chambers et al., 2008; Jha et al., 2007; van den Hurk et al., 2010; Zanesco et al., 2013) and other aspects of EF required to sustain attention such as inhibitory control (Chan & Woollacott, 2007), cognitive flexibility (Moore & Malinowski, 2009), and other cognitive functions in adults and adolescents (Chiesa et al., 2011).

Further, neuroimaging studies have identified brain regions or networks associated with either the affective or the cognitive benefits of mindfulness. Greater dispositional mindfulness was associated with greater correlation between areas of the prefrontal cortex (PFC) and the amygdala in healthy adults (Creswell et al., 2007). Other studies have linked dispositional mindfulness to activation in PFC areas during focused attention and working memory tasks (Dickenson et al., 2013; Stein et al., 2022). Similar findings have been observed in neuroimaging studies examining changes in mindfulness. Mindfulness-training reduction in stress and negative affect has been associated with reduced amygdala activation to emotional stimuli (Bauer et al., 2019; Goldin & Gross, 2010; Lutz et al., 2014; Taylor et al., 2011), and changes in amygdala - ventromedial prefrontal cortex (VMPFC) connectivity (Bauer et al., 2019; Kral et al., 2018). Mindfulness-training enhancement in cognition has been associated with greater activation in PFC areas during inhibitory control (Allen et al., 2012) and lesser resting-state functional connectivity (or greater anti-correlation) between the central executive network (CEN) and the default mode network (DMN) (Bauer et al., 2020).

A fundamental question is whether the benefits of mindfulness for affect and cognition reflect a unitary process or two partly dissociable processes. If mindfulness supports a unitary-regulatory process that enhances control of both cognition and affect (as hypothesized by Glomb et al., 2011; Ma & Fang, 2019; Ryan & Brown, 2003; Tang et al., 2015), the benefits of mindfulness may occur through a unitary “top-down” regulation process, and mindfulness-related variation could be isolated in a single brain area or circuit that explains both affective and cognitive outcomes (Chiesa et al., 2013). If mindfulness exerts its influence on affect and cognition through two partly dissociable or parallel processes, one cannot isolate mindfulness-related variation to a single brain area or circuit to explain both affective and cognitive outcomes. Because most mindfulness studies have focused on either affect or cognition – but not both – it is presently unknown whether the positive effects of mindfulness reflect a unified or distinct dissociable processes.

The present study examined the relationships between mindfulness, affect, cognition, and emotion regulation in an adolescent population using both behavioral and brain measures. Participants were part of a mindfulness study, 40 of whom also participated in neuroimaging. Here we report on behavioral measures along with a reanalysis of previously reported neuroimaging relating mindfulness to either affect (negative affect, stress) (Bauer et al., 2019) or cognition (sustained attention) (Bauer et al., 2020). While the prior two neuroimaging studies focused on the separate effects of mindfulness *training* on affect or cognition, the current study merged the two areas of work to explore the effects of *dispositional* mindfulness on affect and cognition examining whether the effects are dissociable or reflect a shared underlying mechanism.

## Methods

### Participants

Ninety-nine sixth-graders (M_age_ = 12.71 years; 58% male) at the Boston Collegiate Charter school enrolled in a mindfulness study. The study complied with the 1975 Declaration of Helsinki and was approved by the Massachusetts Institute of Technology Committee on the Use of Humans as Experimental Subjects. Guardians gave written informed consent for their adolescent children to participate, and adolescents gave written informed assents for their participation. Approximately 50% of the students had ever been enrolled in the Free/Reduced Price Lunch (FRPL) program for low-income families. The students were 25% Hispanic, 30% African American, 42% White, and 3% other or had multiple racial identities.

All adolescents were invited to participate in the optional neuroimaging substudy of whom 40 volunteered and completed the imaging protocol. The imaging study complied with the 1975 Declaration of Helsinki and was approved by the Massachusetts Institute of Technology Committee on the Use of Humans as Experimental Subjects (see Bauer et al., 2019, and Bauer et al., 2020, for additional details about the study and the analytical approaches to the neuroimaging data). Participants in the imaging substudy did not differ significantly in characteristics from participants who did not participate in imaging (age, gender, racial background, perceived stress, positive and negative affect, and Sustained Attention to Response Task (SART) measures, all *p*s > .11).

### Measures

The inital assessments formed the basis of the behavioral analyses.

#### Perceived stress

Self-perceived stress was measured by the Perceived Stress Scale (PSS) (Cohen et al., 1983; Karam et al., 2012), which asks youth to appraise recent events in their lives as more or less stressful and uncontrollable. The 10-item PSS has been validated in diverse samples, and has strong internal reliability (Cronbach’s α = 0.84; Karam et al., 2012). Items were ranked on a 5-point Likert scale from 0 (never) to 4 (very often) and included items such as: “in the last month, how often have you been upset because of something that happened unexpectedly?” Higher scores on the PSS indicate more frequency of feelings of recent stress.

#### Positive and negative affect

Positive affect (PA) and negative affect (NA) were assessed through the two subscales of the Positive and Negative Affect Schedule for Children (PANAS-C) (Giacomoni & Hutz, 2006). This ten-item shortened version of the original 27-item PANAS-C had previously been validated and used for youth across age ranges (Ebesutani et al., 2012). The PA scale has a Cronbach’s α = .86 and the NA scale has a Cronbach’s α = .82 (Ebesutani et al., 2012). The PA subscale consists of five items (joyful, cheerful, happy, lively, proud) and the NA subscale consists of five items (miserable, mad, afraid, scared, sad). Adolescents self-reported to what degree they had felt that emotion during the past few weeks using a 5-point Likert scale from 1 (very slightly or not at all) to 5 (extremely). Higher scores on each scale indicate more feelings of positive or negative affect.

#### Sustained attention

We measured attentional characteristics through the Sustained Attention to Response Task (SART) (Robertson et al., 1997). The SART is a Go/No-Go task with a high probability of “Go” signals. The SART paradigm was programmed using PsychoPy (Peirce, 2007), a python library for conducting psychological experiments. Participants were instructed to withhold responses (i.e., not pressing space bar) for the number 3 (“No-Go”) and to respond quickly for all other numbers (“Go”). Participants were asked to focus equally on speed and accuracy. Participants could respond either during the stimulus display or during the inter-trial interval (ITI). Participants performed a practice block consisting of 172 Go and No-Go trials, immediately followed by the experimental session consisting of two series of 280 individual digits in which 28 trials or 5% of all trials were No-Go. Digits were presented for 250 ms each with an ITI of 900 ms between each digit. Trial order was pseudo-randomized so that No-Go trials were always separated by at least two Go trials. Participants had the option of an undefined break (not exceeding 5 min) before starting the second series. The task took approximately 15 min to complete. The primary outcome of the SART was accuracy on the “Go” trials, an index of sustained attention, and calculated as the percentage of correct responses (i.e., pressing for numbers 0–9 except for 3) out of all “Go” trials possible (Smilek et al., 2010). Accuracy on “No-Go” trials is an index of response inhibition (correct withholding of a response) (McVay & Kane, 2009; Smallwood, 2013) and was calculated as the percentage of correctly withheld responses to the number 3. Speed of response was measured by average response time (RT) for responses to correct trials only. RTs below 100 ms were removed before calculation of accuracy and average RT.

#### Emotion regulation

Emotion regulation was assessed with a revised version of the Emotion Regulation Questionnaire (ERQ; Gross & John, 2003) for use with children and adolescents (the ERQ-CA). The ERQ-CA comprises ten items assessing the strategies of cognitive reappraisal and expression suppression. The ERQ has been reported to have moderate internal consistency (α = .79 for reappraisal, α = .73 for suppression) as well as sound convergent and discriminant validity with both younger and older adults (Gross & John, 2003). Items were rated on a 5-point Likert scale from 1 (strongly disagree) to 5 (strongly agree), and included items such as: “I control my feelings by not showing them.” Higher scores on each scale (reappraisal and suppression) indicate greater use of the corresponding emotion regulation strategy.

#### Mindfulness

Dispositional mindfulness was measured with the validated six-item scale adapted from the original 15-item Mindful Attention Awareness Scale (MAAS) (Black et al., 2012). This six-item scale has similarly strong reliability when administered to adolescents as when administered to adults (Cronbach’s α = 0.89–0.93), stong consistency, good test-retest reliability (α = 0.89-0.93), and incremental validity for predicting psychopathology in adolescents even when controlling for other psychosocial factors. Items were ranked by a 7-point Likert scale from 1 (almost never) to 7 (almost always), and included questions such as: “I rush through activities without being really attentive to them” (reverse coded). Higher scores on the MAAS indicate greater mindfulness.

### Neuroimaging measures

Forty adolescents both completed the behavioral assessments (described above) and volunteered to participate in the following neuroimaging protocols.

#### Imaging protocol: Resting state

Participants underwent a 6-min resting state scan where they were instructed to passively view a fixation cross during the scan period and not to close their eyes, sleep, or engage in any exercises including mindfulness. Specific instructions were: “Keep your eyes open, relax, try not to move and try to stay awake.” All scans were acquired using a 3T Trio MR System with a 32-channel, phased-array head coil (Siemens Healthcare, Erlangen, Germany). For further details please refer to Bauer et al. (2020).

#### Imaging protocol: Face-matching task

Participants completed a simple matching task paradigm during fMRI in order to measure regional changes in blood flow as a correlate of amygdala activation during the perceptual processing of fearful, happy, and neutral facial expressions and objects (Hariri et al., 2000). On each trial, participants viewed three images on the screen and were asked to select which of the two bottom-row images was identical to the top-row target image. There were four different types of stimuli: fearful faces, happy faces, neutral faces, and objects (fruits and vegetables), and also a resting condition with a fixation cross. There were two runs, with three blocks of each type of stimulus and three fixation blocks per run. Each block consisted of six trials, each presented for 3 s; fixation blocks were 24 s long. Each run lasted 4 min and 48 s. Block order was counterbalanced across participants. For further details please refer to Bauer et al. (2019).

### Analytical approach

We estimated Pearson’s *r* correlation coefficients to quantify the associations among mindfulness, affect, cognition, and emotion regulation. We adjusted the *p*-values using the Benjamini-Hochberg procedure to address multiple hypothesis testing and minimize Type 1 error rate (Benjamini & Hochberg, 1995). We then expanded on these correlations by examining the relationship between mindfulness and affect through a series of multivariate regression models by incrementally adding individual measures of affect and performance on the sustained attention task to each regression. Perceived stress was used as the affective dependent measure as it provided the most parsimonious sequence of regression models and best mirrored the neuroimaging analyses. The primary interest of these regression models is the coefficient for mindfulness, which indicates whether a student’s self-reported mindfulness provided additional predictive information on perceived stress, *above and beyond* what is provided by the other affective and cognitive measures. Because perceived stress, mindfulness, and positive and negative affect are expected to be correlated, these regressions allow us to understand whether these measures (i.e., mindfulness) explain independent variation in perceived stress when controlling for other related measures (i.e., positive and negative affect). This should indicate whether mindfulness is jointly or independently associated with perceived stress and cognitive function. Notably, we also included a covarite of student eligibility for a free and reduced-priced lunch as a measure of socioeconomic status (SES) to ensure our results are robust to the variation in SES in our sample. Because results did not differ with the inclusion of this covariate, we present the most parsimonious models in our results.

As the basis for our neuroimaging analyses, our prior work uncovered two brain functions that were found to change as a consequence of a mindfulness training intervention compared to an active control intervention. For resting state, mindfulness training led to sustained DMN-CEN anticorrelations (Bauer et al., 2020). For the face-matching task, mindfulness training led to decreased right amygdala activations to fearful versus neutral faces (Bauer et al., 2019). In the present study, we investigated whether these processes in the brain were separable by analyzing individual differences in these processes at *baseline,* correlating the brain-based measures with measures of affect (i.e., stress, negative affect) and cognition (i.e., sustained attention). For resting state, we examined the relation of DMN-CEN anticorrelation to baseline variation in SART Go-Accuracy and PSS and NA scores. For Face Matching, we examined the relation between the right amygdala activation to fearful > neutral faces to baseline variation in SART Go-Accuracy and PSS and NA scores. If mindfulness has dissociable brain mechanisms for affective and cognitive processes, we expected that each neural measure would be exclusively associated with only affect (i.e., stress, negative affect) or cognition (i.e., SART Go-accuracy).

## Results

### Bivariate correlational analyses

Greater mindfulness correlated significantly with less perceived stress, less negative affect, more positive affect, and better accuracy on SART (Table 1). Increased use of reappraisal (ERQ-R) correlated significantly with more positive affect, lower negative affect, and less perceived stress, but was unrelated to mindfulness and cognition. However, there were no significant correlations between suppression (ERQ-S) and any of the affective, mindfulness, or cognitive measures. There were no significant correlations between any affective measure (negative affect, positive affect, or perceived stress) and accuracy on SART, nor was there a significant correlation between mindfulness and emotion regulation (reappraisal and suppression), undermining the possibility that mindfulness impacts affect and cognition through emotion regulation as a unitary regulation process.

**Table 1:** Means, standard deviation and Pearson correlation matrix for continuous variables (*N* = 99)

	M	SD	1. PA	2.NA	3.PSS	4. ERQ-S	5. ERQ-R	6. MAAS	7. SART
1. PA	18.53	5.11	-						
2. NA	9.46	4.27	-0.27**	-					
3. PSS	17.12	7.77	-0.42**	0.71**	-				
4. ERQ-S	10.67	3.31	-0.07	0.10	0.15	-			
5. ERQ-R	18.34	5.24	0.58*	-0.28*	-0.37*	0.03	-		
6. MAAS	25.77	7.38	0.24*	-0.43**	-0.50*	-0.05	0.22	-	
7. SART	.86	0.08	0.11	-0.03	-0.11	-0.20	0.07	0.24*	-

All variables are at baseline

1. Positive Affect; 2. Negative Affect; 3. Perceived Stress Scale; 4. Emotion Regulation Questionnaire (S- suppression, R- reappraisal); 5. Mindful Attention Awareness Scale; 6. Sustained Attention to Response Task

Adjusted *p*-values using the Benjamini-Hochberg procedure

*p < 0.05, **p < 0.01, ***p < 0.001

### Regression analyses

A three-step multiple linear regression analysis was performed to evaluate the relationships between mindfulness, perceived stress, positive and negative affect, and cognitive function (SART). Perceived stress was used as the affective dependent measure (see Table 2):

- Model 1: The relationship between mindfulness and perceived stress.
- Model 2: We added positive and negative affect to Model 1 to compare the independent predictive power of mindfulness, negative affect, and positive affect on perceived stress. Associations between mindfulness and measures in the affective domain (i.e., perceived stress, positive and negative affect) would suggest one affective process related to mindfulness.
- Model 3: We added cognitive function to Model 2 to examine its association with perceived stress when controlling for positive and negative affect. Associations between mindfulness and cognition (ref. Table 1) along with mindfulness and perceived stress but not cognition and perceived stress as seen here through Model 3 would suggest two dissociable processes related to mindfulness.

**Table 2:** Regression model of the relationship between perceived stress and mindfulness

	Model 1		Model 2		Model 3	
Variable	*B* (SE)	β	*B* (SE)	β	*B* (SE)	β
Mindfulness	-0.53***	-0.5	-0.21**	-0.2	-0.23**	-0.22
	(0.09)		(0.07)		(0.07)	
Positive Affect			-0.34**	-0.22	-0.34***	-0.23
			(0.10)		(0.10)	
Negative Affect			1.02***	0.56	0.96***	0.54
			(0.13)		(0.13)	
SART-Go Accuracy					-1.54	-0.01
					(6.09)	
Adjusted R^2^	0.25		0.59		0.59	
N	99		99		99	

*p < .05, **p < .01, ***p < .001

### Mindfulness and affect

In Model 1, a linear regression was calculated to predict perceived stress based on mindfulness. The multivariate regression replicates the relationship that we observed with the Pearson correlation in Table 1. A one-standard deviation increase in mindfulness predicted a 0.5-standard deviation decrease in perceived stress (β = -0.5, *B* = 0.53, t = - 5.75, *p* < 0.001, CI [-0.714, -0.34], BF_10_ = 111,611). This model accounted for a moderate amount of variance in perceived stress (F(1,97) = 33.03, *p* < 0.001, R^2^_adjusted_ = 0.25).

In Model 2, negative and positive affect were added to the regression model. When we include negative and positive affect, the association of mindfulness with perceived stress decreased, suggesting omitted variable bias, but still remained independently predictive of perceived stress (β = -0.2, *B* = -0.21, t = -2.78, *p* = 0.006, CI [-0.36, -0.06], BF_10_ = 111,611), while controlling for affect (negative and positive). A one-standard deviation increase in mindfulness predicted a 0.2-standard deviation decrease in perceived stress. Adding negative and positive affect enhances the explained variance in perceived stress (F(3, 95) = 47.29, *p* < 0.001, R^2^_adjusted_ = 0.59).

### Mindfulness, affect, and cognitive function

In Model 3, we added cognitive function to Model 2. The bivariate correlation analyses indicated that mindfulness and cognitive function were significantly correlated but that stress and cognitive function were not. To confirm this null relationship, we included cognitive function as a predictor in the regression model (Model 3), which confirmed no relationship between cognitive function and perceived stress (β = -0.01, *B* =-1.54, t = -0.25, *p* = 0.80, CI [-13,64 – 10.55], BF_10_ = 0.38). Moreover, the association between mindfulness and positive and negative affect as well as the R^2^ were relatively unchanged from Model 2, which indicates that the cognitive measure is not a key omitted variable. The inclusion of Model 3 confirms that stress and cognitive function were unrelated.

To assess whether these analyses were sufficiently powered, we used the G*Power software package to compute a post hoc power analysis (Faul et al., 2007) that indicated our sample of *n* = 99 in the regression model with four predictors with the smallest effect size of .22 and ⍺ < .05 achieved power at 97%.

### Imaging analyses

#### Relation of PSS and NA scores to DMN-CEN anticorrelation

There were no significant correlations between DMN-CEN functional connectivity and either PSS scores (*r* = -.1, *n* = 31, *p* = .27) or negative affect (r = .04, *n* = 31, *p* = .58). This is in contrast to a significant negative correlation between DMN-CEN connectivity and SART accuracy (r = -.45, *n* = 31, *p* = 0.005, FWE-corrected) (Bauer et al., 2020).

### Relation of SART accuracy to right amygdala activation

There was no significant correlation between SART Go-accuracy and right amygdala activation (*r* = .04, *n* = 31, *p* = .79). This is in contrast to the significant correlations between amygdala activation to the fear > neutral face matching contrast and PSS scores (*r* = 0.41, *n* = 39, *p* = 0.02, FWE-corrected) and negative affect (*r* = 0.45, *n* = 39, *p* = 0.03, FWE-corrected) (Bauer et al., 2019).

An a priori power analysis was conducted to determine the minimum sample size required to test the neuroimaging study hypothesis. For a within-subject condition with a repeated-measures ANOVA at the small effect sizes, G*Power software indicated a sample of *n* = 18 was required for each group to achieve sufficient power (80%) to detect a small effect size at ⍺ < .05.

## Discussion

The present study examined the relationship between dispositional mindfulness and both affective and cognitive well-being in an adolescent population. Using behavioral and brain measures, our findings suggest that the relationships between dispositional mindfulness and affective and cognitive processes are partly dissociable. These exploratory findings do not support the alternative hypothesis that mindfulness has a unitary influence on affect and cognition, but do offer novel evidence on the dissociable relations between mindfulness and affect or cognition.

The behavioral findings further solidify the relationship between mindfulness and affective well-being: Greater dispositional mindfulness was related to less perceived stress, less negative affect, and more positive affect. Moreover, regression analyses revealed that mindfulness remained independently predictive of perceived stress even after controlling for negative and positive affect. These findings align with a broader literature that finds a positive link between dispositional mindfulness and emotional well-being (Bajaj et al., 2016; Barnhofer et al., 2011; Brown et al., 2012; Fossati et al., 2011; Hou et al., 2015; Smith et al., 2011).

Similarly, the finding that greater mindfulness was associated with better sustained attention corroborates the link between mindfulness and cognition. Meta-analyses indicate that greater mindfulness is associated with better attention and EF in youth and adults (Cásedas et al., 2020; Chiesa et al., 2011; Dunning et al., 2022; Lao et al., 2016; Leyland et al., 2019; MacCoon et al., 2014; Yakobi et al., 2021). Despite this positive evidence, a review of mindfulness research in youth found five of 13 studies showed medium to large positive effects on attention and EF, but eight studies failed to detect this positive relation (Mak et al., 2018). Given our use of one measure of EF (i.e., sustained attention), it is unclear whether the diverse findings in the broader literature relate to the variability of how attention and EF are assessed across studies, and whether trait mindfulness is more related to some of these functions.

The present findings extend prior research by indicating a possible dissociation between the affective and cognitive benefits of mindfulness. First, the behavioral results demonstrate that affective and cognitive measures were related to mindfulness but not to each other. Specifically, sustained attention was unrelated to perceived stress (regression analyses Model 3), and emotion regulation was uncorrelated to mindfulness. Interpreting non-significant findings as evidence for the null hypothesis can be challenging, but following the suggested guidelines by Dienes (2014) for null hypothesis significance testing, three metrics support the null hypothesis that the affective and cognitive values were unrelated: (1) the analyses were all sufficiently powered; (2) the confidence interval demonstrates that cognitive function was not related to perceived stress; and (3) the Bayes factor for the cognitive variable (BF_10_ = 0.38) in the regression model favors the null hypothesis (note that BF_10_ values < 1 are in favor of H_0_ over H_1_). Neuroimaging analyses also corroborated these behavioral findings: Brain connectivity associated with SART accuracy was uncorrelated with affective measures, whereas brain activation associated with perceived stress was uncorrelated with SART accuracy. Collectively, these findings provide compelling preliminary evidence that the effects of mindfulness on affect and cognition occur at least partially in an independent manner, rather than as a unitary process.

We identified two distinct brain mechanisms. First, variation in affect was associated with amygdala activation during a face-matching task. Mindfulness involves paying attention to affective stimuli and experiences without trying to control them. This “implicit” regulation of affect may dampen reactivity in brain structures like the amygdala (Guendelman et al., 2017). Previous studies found reduced amygdala responses to emotional face distractors related to increased mindfulness in female adolescents (Dumontheil et al., 2023), as well as negative correlations between EEG emotional responses and the observing subscale of the Five Facet Mindfulness Questionnaire (Deng et al., 2021). These findings have not, however, been replicated in other clinical populations of youth (Strawn et al., 2016). Mindfulness may have its positive impacts on affect through changes in emotional responses in the brain through decreased reactivity.

Second, variation in cognition (SART accuracy) was associated with connectivity of the DMN-CEN networks at rest. Lesser resting-state functional connectivity (stronger anti-correlations) between the DMN and CEN has been associated with better working memory (Hampson et al., 2010; Keller et al., 2015), sustained attention (Kucyi et al., 2020), and performance on cognitive tests (Ellwood-Lowe et al., 2021). Because mindfulness involves more present-moment awareness and focused attention (as opposed to mind wandering), mindfulness may involve decoupling the DMN and CEN networks (Hellyer et al., 2014; Mooneyham et al., 2017). Previous studies have found mindfulness-related differences in connectivity of large-scale networks like the DMN, CEN, and the salience network (SN), but not necessarily decoupling of the DMN and CEN. States of anticorrelated DMN-SN connectivity are more common in higher trait mindfulness in youth (Marusak et al., 2018) and in another study, DMN-CEN connectivity during a working memory task negatively correlated with the nonreactivity scale of the adult and adolescent mindfulness scale (Stein et al., 2022).

The dissociation between mindfulness relations with emotion and cognition can be considered in the context of other neurocognitive dissociations in the brain. For example, there is a fundamental dissociation between ventral (“what”) and dorsal (“where”) visual streams (Ungerleider & Haxby, 1994) and also between declarative (“knowing what”) and procedural (“knowing how”) memory systems (Cohen & Squire, 1980). However, the two kinds of processes often interact to support high-level cognition. Similarly, the dissociation between affect and cognition found in the present study illuminates the mechanistic separability of these processes in relation to mindfulness, but that dissociation does not preclude interactions (and thus associations) between these processes. In fact, a growing literature indicates that affect and cognition are intimately interconnected (Barrett & Satpute, 2013; Immordino-Yang & Damasio, 2007; Okon-Singer et al., 2015). Even the link between greater mindfulness and better academic outcomes may be generated by the intersection of improved cognitive and affective processes (Caballero et al., 2019).

Our findings shed light on the separability between trait mindfulness and affective versus cognitive processes in the mind and brain, but limitations of the present discovery ought to be addressed in future research. The MAAS was used given its extensive use and strong validity and reliability as a measure of mindfulness (e.g., Black et al., 2012), but it focuses on a single dimension of mindfulness. Mindfulness may be a multifaceted construct, and future research could explore whether different facets of mindfulness, as measured by the Five Facet Mindfulness Questionnaire (FFMQ; Baer et al., 2006) for instance, relate to different behavioral and brain pathways. Similarly, the use of single measures for emotion regulation and for cognition also constrained our ability to fully test against the unitary hypothesis. Emotion regulation is a candidate for involving an interaction between affective and cognitive benefits of mindfulness, but the measure of emotion regulation used as a proxy of a unitary process in the present study (ERQ-CA) was only partially correlated with some measures. While the ERQ-CA has demonstrated construct validity and is commonly used (Gullone & Taffe, 2012), our inability to detect its expected associations with other measures raises concerns of the questionnaire as a comprehensive and sufficiently sensitive measure of emotion regulation. Although our findings did not capture relations between any form of emotion regulation (suppression or reappraisal) and accuracy on SART, another study found that EF mediated the effect of mindfulness on emotion regulation (Teper et al., 2013). Similarly, the SART was selected as the behavioral measure of cognition given the strong relationship between self-reported mindfulness and performance on sustained attention tasks (Schmertz et al., 2009), but future work would be better served by including other measures of sustained attention and EF to examine the relationship between mindfulness, emotion regulation, and cognition. Nonetheless, our multi-modal approach in combining behavioral, self-report, and neuroimaging data yields converging evidence in favor of some dissociation between psychological and neurobiological mechanisms underlying the relation of trait mindfulness to cognitive versus affective processes.

The present findings are exploratory but have important implications for supporting the well-being of developing adolescents. Stress exposure is increasingly common for youth, particularly at-risk youth, and empirical findings emphasize its deleterious effects on both mental health and learning outcomes (Tsai et al., 2020). The benefits of mindfulness are both affective and cognitive, and mindfulness is malleable. This analysis examined dispositional mindfulness and did not examine intentional mindfulness that follows mindfulness training or long-term practice (Wheeler et al., 2017). Future research could examine whether these findings extend to intentional or trained mindfulness aimed at enhancing affective and cognitive well-being in adolescents (e.g., Bauer et al., 2019, 2020).
